# Endoscopic submucosal dissection to treat squamous cell carcinoma in situ of the anal canal

**DOI:** 10.1016/j.vgie.2022.02.011

**Published:** 2022-04-06

**Authors:** Michael Lajin, Mohamed O. Othman, Rokay Kamyar, Octavio Armas

**Affiliations:** 1 SHARP health, San Diego, California; 2 Baylor College Of Medicine, Houston, Texas

**Keywords:** AIN, anal intraepithelial neoplasia, ESD, endoscopic submucosal dissection, HSIL, high-grade squamous intraepithelial lesion

## Abstract

**Background and Aims:**

The standard treatment for invasive squamous cell anal cancer is chemoradiation treatment. However, treatment options for high-grade dysplasia (squamous cell cancer in situ) are either surgical excision or topical treatment modalities. There are a few case reports, mainly from Japan, about resecting early squamous cell anal cancer (high-grade dysplasia/carcinoma in situ) by endoscopic submucosal dissection. We present a case series of 3 patients from a western hemisphere population with squamous carcinoma in situ of the anal canal resected with endoscopic submucosal dissection (ESD).

**Methods:**

This is a retrospective series of 3 patients from a western hemisphere population with squamous carcinoma in situ of the anal canal resected with ESD. All patients were referred from the oncology team after declining surgical excision.

**Results:**

Microscopically margin-negative en bloc resection was achieved in all patients. All patients were free from dysplasia or cancer on their latest endoscopic surveillance, ranging from 10 months to 26 months after ESD. One patient had a small lesion on follow-up 3 months after ESD that was removed by a curative EMR. There were no immediate or delayed adverse events.

**Conclusions:**

ESD can be used to resect squamous cell carcinoma in situ of the anal canal. Larger studies with long-term follow-up are needed to evaluate the role of ESD in early squamous cell anal cancer and to compare it with other modalities of treatment.

## Introduction

Squamous cell carcinoma is the most common cancer involving the anal canal, with an incidence of 1.8 per 100,000. It originates from squamous dysplastic lesions associated with human papillomavirus, particularly subtype 16. Risk factors include HIV, men who have sex with men, history of cervical cancer, and renal transplant patients.

The Lower Anogenital Squamous Terminology standardization project classifies squamous dysplastic lesions into 2 categories: low-grade squamous intraepithelial lesion, which correlates with anal intraepithelial neoplasia (AIN) 1 (anal condyloma), and high-grade squamous intraepithelial lesion (HSIL), which correlates with AIN 2-3.^1^

Although chemoradiation therapy is the standard treatment for invasive squamous cell cancer of the anal canal,^2^ there is significant controversy regarding the management of HSIL/AIN-3 lesions (squamous cell cancer in situ). Treatment modalities for HSIL/AIN-3 lesions include surgical excision and ablative/topical therapies.

Endoscopic submucosal dissection (ESD) is being increasingly used to manage precancerous and early cancerous lesions in the GI tract. Multiple case reports from Japan have demonstrated the efficacy of ESD in treating HSIL/AIN-3 lesions.^3^, ^4^, ^5^, ^6^, ^7^, ^8^, ^9^

We present a case series of 3 patients from a Western population with HSIL/AIN-3 lesions managed with ESD.

## Patients and methods

### Patient 1

**Figure 1:**
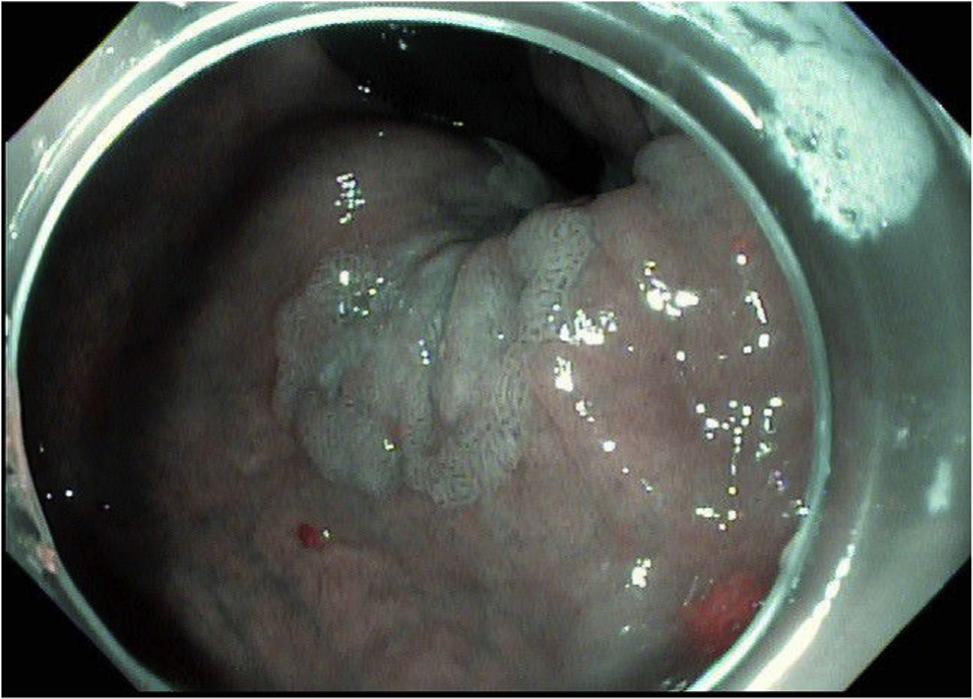
Case 1: Pre–endoscopic submucosal dissection narrow-band image of anal intraepithelial neoplasia-3 lesion.

A 76-year-old woman with a history of diabetes was found to have a 3-cm, white, flat, elevated lesion involving the distal rectum and the anal canal (Fig. 1). Narrow-band imaging showed irregular vascular patterns (dilation, tortuosity, irregular shapes, and caliber changes). A biopsy specimen was consistent with “a high-grade squamous intraepithelial lesion (AIN-3).” She was evaluated by oncology and was referred for endoscopic resection after declining surgical excision.

**Figure 2:**
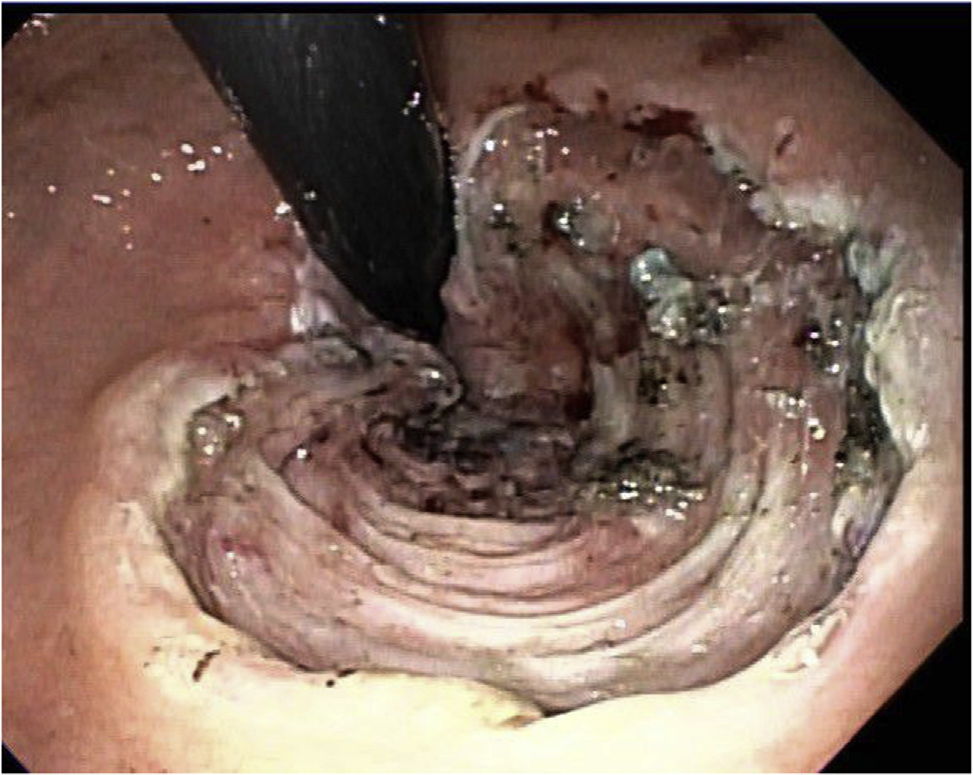
Case 1: Post–endoscopic submucosal dissection endoscopic image.

The lesion was marked, and a submucosal injection was performed using ORISE Gel (Boston Scientific, Marlborough, Mass, USA). A circumferential mucosal incision was performed using a dual knife (endo cut current) (Olympus, Shinjuku City, Tokyo, Japan). Submucosal dissection was performed using the same knife with a forced coag current. The final submucosal dissection was facilitated by an IT knife (Olympus) with forced coag current (Fig. 2). The specimen was retrieved in 1 piece, measuring 4.7 × 2 cm. There were no immediate or delayed adverse events.

**Figure 3:**
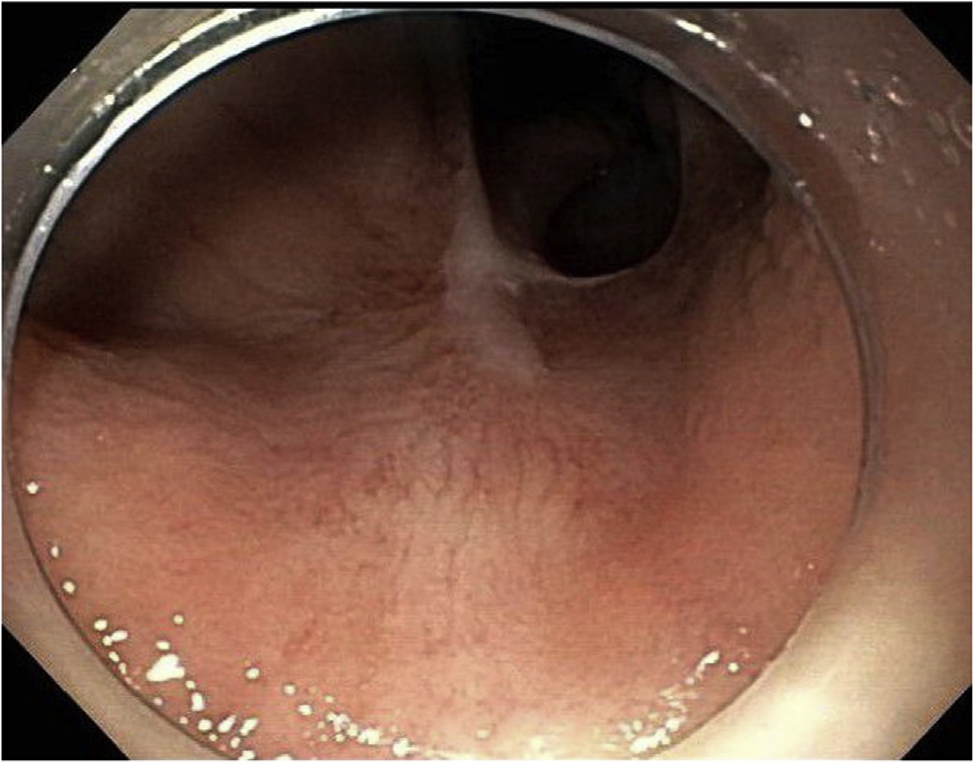
Case 1: Follow-up image after 10 months.

The final pathology was consistent with a high-grade squamous intraepithelial lesion (AIN-3). The margins were negative for dysplasia. Follow-up endoscopic evaluation at 3 months and then at 10 months (Fig. 3) showed no dysplasia/carcinoma.

### Patient 2

**Figure 4:**
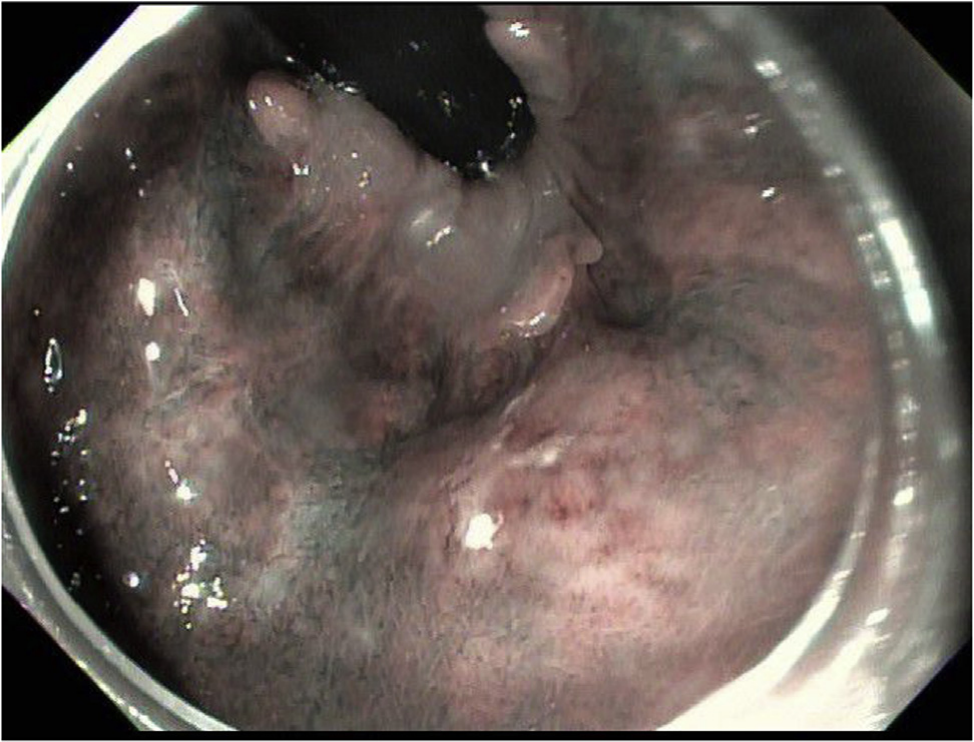
Case 2: Pre–endoscopic submucosal dissection narrow-band image of anal intraepithelial neoplasia-3 lesion.

A 71-year-old woman was found to have a 1.5-cm, white, flat, elevated lesion involving the distal rectum and anal canal (Fig. 4). A biopsy specimen was consistent with “fragments of squamous carcinoma in situ.” She was evaluated by oncology and was referred for endoscopic resection after declining surgical excision.

**Figure 5:**
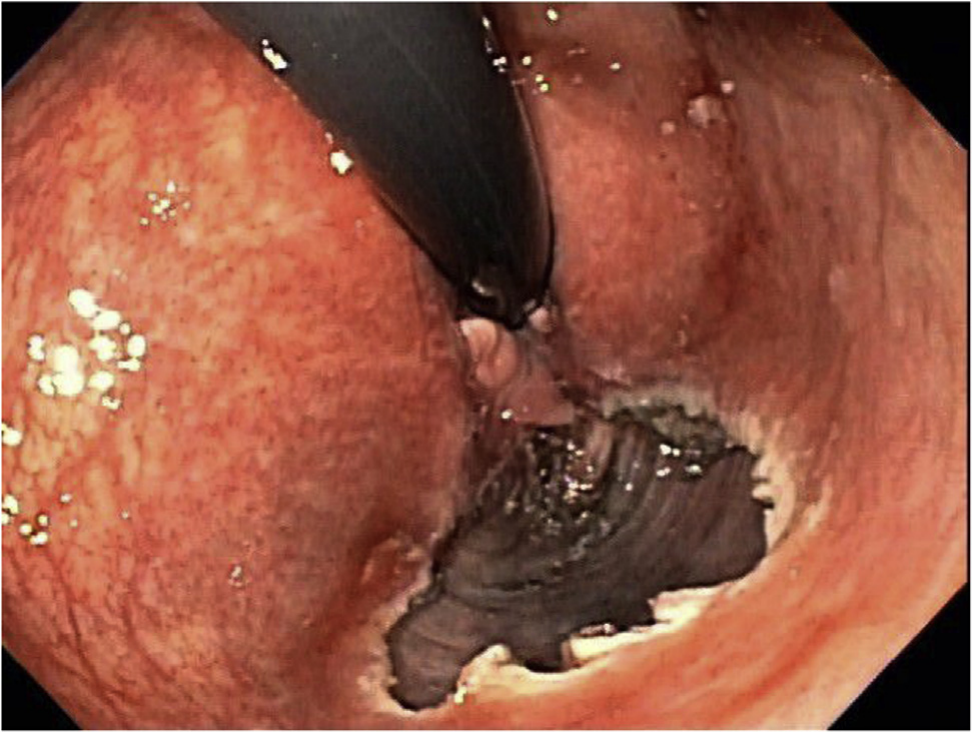
Case 2: Post–endoscopic submucosal dissection endoscopic image.

The lesion was marked, and a submucosal injection was performed using ORISE Gel. The pocket technique was used. A mucosal incision was performed using a dual knife with endo cut current caudal to the lesion. The gastroscope entered a submucosal tunnel. Submucosal dissection was performed using the same knife with a forced coag current. After completing the dissection underneath the lesion, the circumferential mucosal incision was completed using an SB knife (Olympus) with endo cut current (Fig. 5). The specimen was retrieved in 1 piece, measuring 3.1 × 2.3 cm. There were no immediate or delayed adverse events.

**Figure 6:**
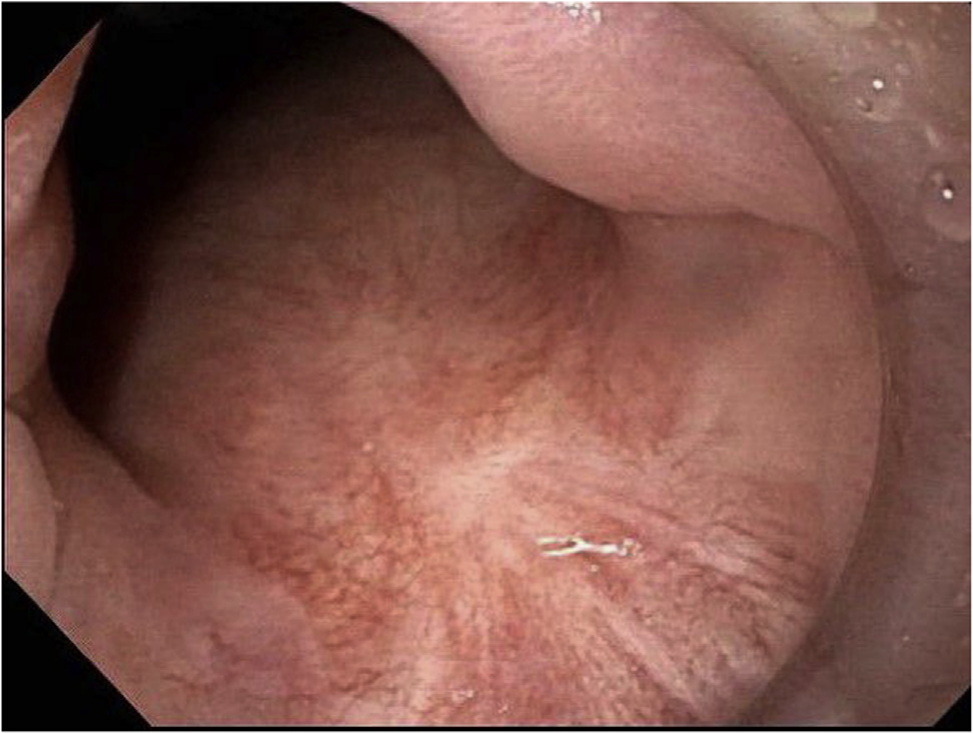
Case 2: Follow-up image after 26 months.

The final pathology was consistent with squamous cell carcinoma in situ. The margins were free from carcinoma/dysplasia. Follow-up endoscopic evaluation at 4, 14, and 26 months (Fig. 6) after ESD showed no dysplasia/carcinoma.

### Patient 3

**Figure 7:**
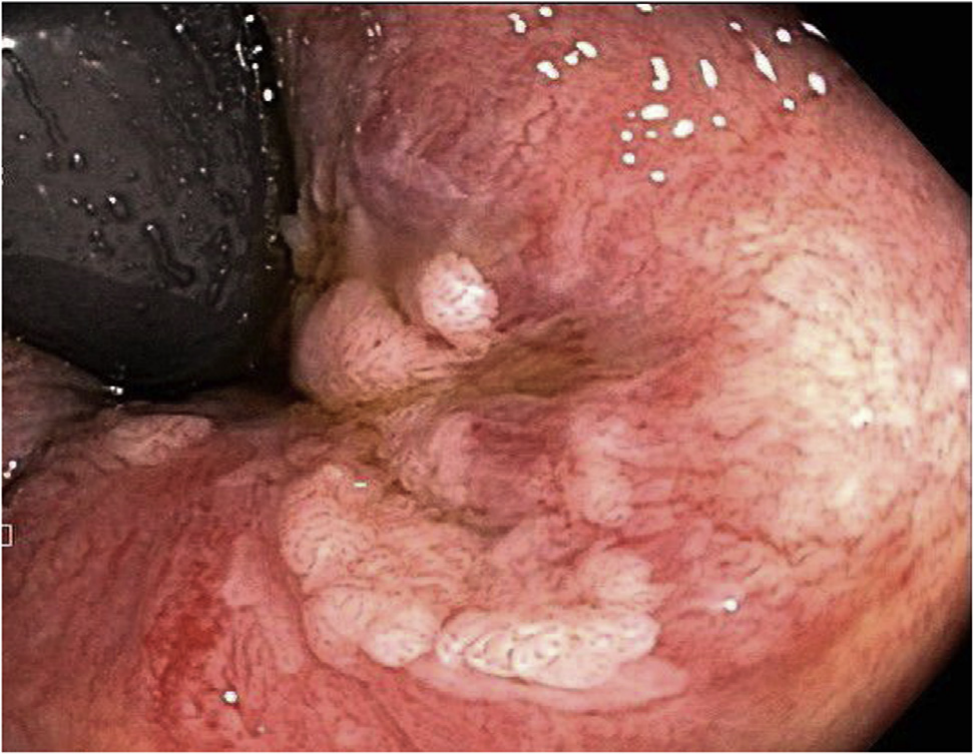
Case 3: Pre–endoscopic submucosal dissection endoscopic image of anal intraepithelial neoplasia-3 lesion.

A 62-year-old man with a history of coronary artery disease and diastolic congestive heart failure was found to have a 3.5-cm, white, flat, elevated lesion involving the distal rectum and the anal canal (Fig. 7). Narrow-band imaging showed irregular vascular patterns (dilation, tortuosity, irregular shapes, and caliber changes). A biopsy specimen was consistent with “a high-grade squamous intraepithelial lesion (AIN-3).” He was evaluated by oncology and was referred for endoscopic resection after declining surgical excision.

**Figure 8:**
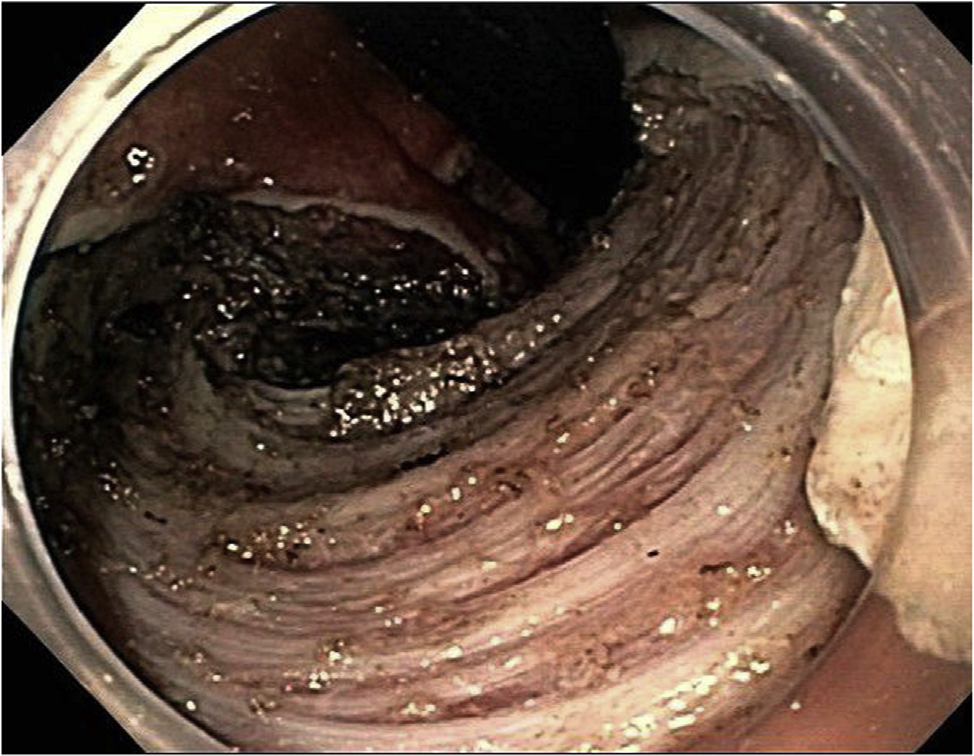
Case 3: Post–endoscopic submucosal dissection endoscopic image.

The lesion was marked, and a submucosal injection was performed using ORISE Gel. The pocket technique was used. A mucosal incision was performed using a dual knife with endo cut current caudal to the lesion. The gastroscope entered a submucosal tunnel. Submucosal dissection was performed using the same knife with a forced coag current. After completing the dissection underneath the lesion, the circumferential mucosal incision was completed using an SB knife with endo cut (Fig. 8). The specimen was retrieved in 1 piece, measuring 5.4 × 4.0 cm. There were no immediate or delayed adverse events.

**Figure 9:**
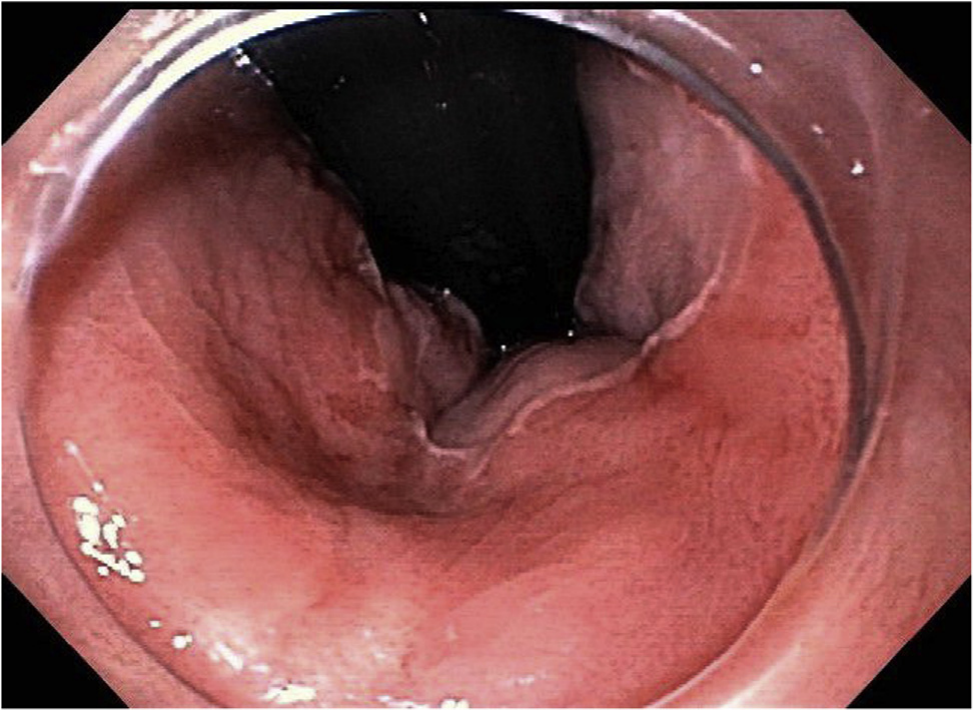
Case 3: Follow-up image after 15 months.

The final pathology was consistent with a high-grade squamous intraepithelial lesion (AIN-3). The margins were negative for dysplasia. Follow-up endoscopic evaluation 3 months later showed an 8-mm lesion located outside the scar. The lesion was removed with band-assisted EMR. The pathology was consistent with AIN-3. The margins of resection were negative. Another endoscopic evaluation was performed 3 months after EMR and showed no residual dysplasia/carcinoma. Repeated endoscopic evaluation 15 months after initial ESD showed no residual cancer/dysplasia (Fig. 9).

## Discussion

Preliminary studies estimate the rate of progression of HSIL/AIN-3 lesions to anal cancer to be 9% to 13% within 5 years.^10^

Surgical excision for these lesions was the preferred treatment in the past. However, the rate of recurrence/incomplete excision remains high at 9% to 63%.^11^ Repeated surgical excisions increase the risk of stenosis or fecal incontinence. Topical treatments such as trichloroacetic acid, imiquimod, and 5-fluorouracil and ablative techniques such as fulguration with electrocautery or coagulation with infrared or laser are less invasive but carry a significant risk of recurrence.^12^ Expectant management and watchful waiting by surveillance alone for HSIL/AIN-3 lesions expose patients to the risks of chemoradiation treatment if they convert to invasive anal cancer.

ESD uses the concept of dissecting the submucosal fibers underneath the lesion to achieve en bloc resection. It is now recognized as a preferred noninvasive treatment modality for premalignant and early malignant GI lesions. ESD allows resection of lesions in anatomically difficult locations such as the anal canal. En bloc resection provides accurate histological assessment to evaluate the adequacy of the treatment.

The key elements of ESD, such as using flexible endoscopy, distal attachment caps, smart electrosurgical units/knives, and accurate markings of borders combined with deep submucosal dissection, lead to a precise resection to decrease the chance of incomplete resection or recurrence. Multiple reports of curative ESD for HSIL/AIN-3 lesions have emerged from Asian countries such as Japan.

We present in this case series 3 patients from a western population who underwent ESD for HSIL/AIN-3 lesions. All 3 patients were referred by our oncology colleagues after declining surgical excision. Both the conventional method and the pocket creation method^13^ were used in this case series.

The lesions in cases 2 and 3 were resected using the pocket creation method. This method allows preservation of the fluid in the submucosal space, resulting in an easier and more efficient dissection, particularly in difficult anatomical locations such as the anal canal. On the other hand, the lesion in case 1 was wider. The conventional method was selected in this case because it enables us to mark off the dissection endpoints at the borders of the lesion.

The initial mucosal incision was started at the anal side. This approach allowed us to perform the initial submucosal dissection with a straight rather than a retroflexed endoscope position. The incision at the anal side is more difficult. Thus, it is preferable to start with detaching that side first.^14^ ORISE Gel was the injection solution used in all cases because of its ability to provide a long-lasting submucosal fluid cushion.

All 3 procedures were performed with the patient under general anesthesia; as a result, lidocaine, which typically provides a transient numbing effect, was not used. Aside from minor rectal discomfort that was well controlled with oral acetaminophen, patients did not experience any significant rectal pain after all ESD procedures.

Successful en bloc resection was achieved in all patients. The ESD pathology was consistent with AIN-3 without invasive cancer. All patients had microscopically margin-negative resection with free margins. One patient was found to have an 8-mm AIN-3 lesion during 3-month follow-up surveillance, despite the negative margins achieved with the initial ESD. Multifocal AIN-3 lesions are reported in the literature.^15^ This lesion was located outside the scar of the previous ESD site; therefore, it is likely a separate focus of AIN-3. Given the small size of the lesion and the absence of scarring, it was removed safely with band-assisted EMR with clear margins.

Currently, all patients are clear from dysplasia/malignancy on their latest endoscopic surveillance ranging from 10 to 26 months after ESD. Because the pathology of the resected lesions is consistent with AIN-3 with free margins, all 3 patients are undergoing regular endoscopic surveillance, and no further treatment is warranted at this time. There were no immediate or delayed adverse events, such as bleeding, perforation, infection, stenosis, or fecal incontinence.

## Conclusions

ESD is a promising noninvasive modality for treating HSIL/AIN-3 lesions. Larger studies with a longer follow-up are needed to assess its role in this clinical setting and to compare it with other modalities of treatment (Video 1, available online at www.giejournal.org).

## Disclosure

*Dr Othman is a consultant for Olympus, Boston Scientific, Conmed, Lumendi, and Apollo and has received research grants from Abbvie, Conmed, and Lucid Diagnostic. All other authors disclosed no financial relationships.*

## Supplementary data

Video 1 Video description of 3 patients with anal intraepithelial neoplasia-3 lesions of the anal canal/distal rectum resected with endoscopic submucosal dissection.
